# Anomalous azygos venous system in a south Indian cadaver: a case report

**DOI:** 10.4076/1757-1626-2-6746

**Published:** 2009-09-14

**Authors:** Lydia S Quadros, Bhagath Kumar Potu, Anitha Guru, Thejodhar Pulakunta, Biswabina Ray, Suhani Sumalatha D'Silva, Sylvia Sequeira, Huban Thomas

**Affiliations:** 1 Department of Anatomy, Kasturba Medical College, Manipal University, Manipal, Karnataka, India; 2 Department of Anatomical Sciences, St. Matthews University School of Medicine, Grand Cayman Islands, Cayman Island, British West Indies; 3 Department of Anatomy, KMC International Centre, Manipal University, Manipal, Karnataka, India

## Abstract

**Introduction:**

The posterior thoracic wall, an area drained by the azygos venous system, is a common site for surgical intervention. Since the venous part of the cardiovascular system is subject to most common variation, abnormalities in the azygos venous system are often reported.

**Case presentation:**

During routine dissection classes for undergraduate medical students, we encountered a variation in the azygos venous system in a 65 years old south Indian male cadaver. We observed that there was no accessory azygos vein, and left 4^th^, 5^th^, 6^th^ and 7^th^ posterior intercostal veins terminated directly into azygos vein.

**Conclusion:**

Identifying these types of variations is important during imaging this region and surgical operations of mediastinum.

## Introduction

The azygos system includes those veins, which are straight in course and not accompanied with the corresponding arteries. The azygos system drains blood from the back and from the thoracic and abdominal walls. Normally, the azygos vein on right side receives all the posterior intercostals veins except the first vein. On the left side, the accessory azygos vein, a tributary of azygos vein receives the blood from left 5^th^, 6^th^ and 7^th^ posterior intercostals veins, where as the left lower four posterior intercostals veins (8^th^, 9^th^, 10^th^ and 11^th^) opens into hemi azygos vein, which is an another tributary of azygos vein [1]. In some cases branching pattern of azygos vein may not be constant like above description. Bergman et al. have reported the incidence of the variant branching pattern of azygos vein was 26% in the cases he dissected [2]. Abnormalities about the accessory azygos and hemiazygos veins are not very common. In this paper, we are reporting the underdeveloped hemiazygos, and direct opening of 4^th^, 5^th^, 6^th^ and 7^th^ left posterior intercostals veins into the azygos vein.

## Case presentation

**Figure 1:**
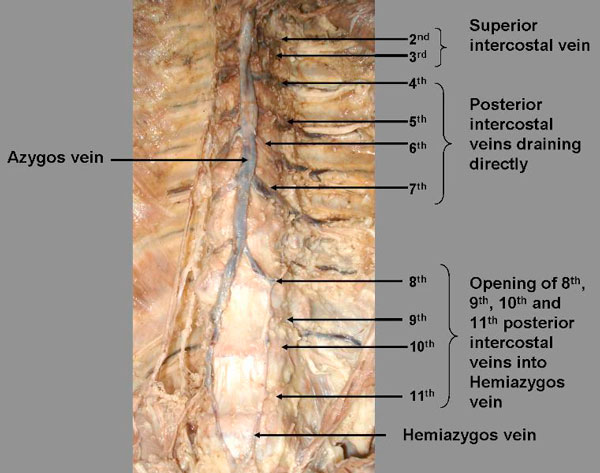
**Showing the abnormal drainage of left 4^th^, 5^th^, 6^th^ and 6^th^ posterior intercostal veins into azygos veins**. We can also see underdeveloped Hemiazygos vein in the picture

During the routine dissection classes for medical students, we observed a variation in the azygos venous system in a 65 years old south Indian male cadaver. It was found that there was no complete accessory hemiazygos vein in the azygos venous system because of its absence, the left posterior intercostal veins (4^th^, 5^th^, 6^th^ and 7^th^) drained directly into the azygos vein. Right ascending lumbal vein formed azygos vein and left ascending lumbal vein formed hemiazygos vein, which is underdeveloped (Figure 1). All other major vessels like superior vena cava and inferior vena cava were absolutely normal.

## Discussion

In this paper, we report the underdeveloped hemiazygos veins and the direct opening of 4^th^, 5^th^, 6^th^ and 7^th^ left posterior intercostal veins into the azygos vein. There are different types of variations were reported on the branching pattern of azygos vein. Ozbek et al. reported that the hemiazygos vein was absent in their case [3]. Cossina et al. have reported two azygos veins that continue with inferior vena cava [4]. These abnormalities are generally explained by the embryological development. Azygos veins embryologically generate from subcardinal veins. The right subcardinal vein forms azygos vein and the left subcardinal vein forms hemiazygos vein. A transverse anastomosis is formed between them at sixth and seventh thoracic vertebrae in adults. At the left side, cranial part of this anastomosis remains as accessory hemiazygos vein. In our case accessory hemiazygos vein did not exist due to total regression of the left subcardinal vein and atrophy of veins forming hemiazygos and accessory hemiazygos veins that were embryologically above anastomosis of right and left post cardinal veins because of this, the left posterior intercostal veins (4^th^, 5^th^, 6^th^ and 7^th^) drained into the azygos vein [5]-[7].

It is very important to identify the variations of the azygos system especially in the computed tomography and magnetic resonance imaging of mediastinum. The abnormal azygos venous system may easily be confused with aneurysm, lymphadenopathy and other abnormalities like tumor [3,8,9]. It is important to keep these kinds of variations in mind while performing the mediastinal operations or surgery of large vessels.

## Consent

Written informed consent was obtained from the subject's relative for publication of this case report and accompanying images. A copy of the written consent is available for review by the Editor-in-Chief of this journal.

## Competing interests

The authors declare that they have no competing interests.

## Authors' contributions

LSQ, AG and TP did the literature search and wrote the case report and also obtained written consent. BKP and BR helped to draft the manuscript. SSD, SS and HT helped in the literature search. All authors had gone through the final manuscript and approved it.
